# microRNA-199a is able to reverse cisplatin resistance in human ovarian cancer cells through the inhibition of mammalian target of rapamycin

**DOI:** 10.3892/ol.2013.1448

**Published:** 2013-07-08

**Authors:** ZHONGXIAN WANG, ZHOU TING, YA LI, GANG CHEN, YUNPING LU, XING HAO

**Affiliations:** 1 Department of Obstetrics and Gynecology, The First College of Clinical Medical Sciences and Yichang Central People’s Hospital, China Three Gorges University, Yichang, P.R. China; 2 Department of Obstetrics and Gynecology, Tongji Hospital, Tongji Medical College, Huazhong University of Science and Technology, Wuhan, Hubei, P.R. China

**Keywords:** microRNA-199a, mammalian target of rapamycin, cisplatin resistance, ovarian cancer cells

## Abstract

microRNAs (miRNAs/miRs) may have a crucial function in tumor metastasis through the regulation of a plethora of signaling pathways. Increasing evidence has shown that miR-199a is important in regulating the tumor metastasis of ovarian cancer, although the precise biological function of miR-199a is unclear at present. In the current study, it was observed that the expression levels of miR-199a were higher in OV2008 cells compared with C13* cells. However, lower levels of mammalian target of rapamycin (mTOR) protein were detected by western blotting in the OV2008 cells compared with the C13* cells. The miR-199a levels were increased in the C13* cells using miR-199a mimics and the mTOR levels were observed to decrease. This may have resulted in a reversal of cisplatin resistance in the C13* cells. To test this hypothesis, the Renilla luciferase reporter gene system was used to analyze the mTOR levels. The results indicated that the expression levels of mTOR were significantly blocked by the increased miR-199a levels. When the miR-199a inhibitor was applied to decrease the miR-199a levels, it was observed that the mTOR expression levels were increased, while cisplatin-induced apoptosis was decreased in the OV2008 cells. The study concludes that miR-199a is able to reverse cisplatin resistance in human ovarian cancer cells through the inhibition of mTOR and that mTOR may be the target of miR-199a during this process.

## Introduction

The American Cancer Society estimated that 21,880 women in the United States would be diagnosed with ovarian cancer and 14,621 of them would succumb to this disease in 2010 (1). The current standard treatment for advanced-stage ovarian cancer is cytoreductive surgery and cisplatin-based combination chemotherapy. However, drug resistance commonly develops following a few cycles of therapy and the mechanism of drug resistance remains unclear. Studies have demonstrated that mammalian target of mammalian target of rapamycin (mTOR) may contribute to this cisplatin resistance (2).

microRNAs (miRNAs/miRs) are post-transcriptional regulators that bind to complementary sequences on target messenger RNA (mRNA) transcripts, usually resulting in translational repression or target degradation and gene silencing (1,3). miR-199a is located on human chromosome 19q13.2 (3) and has been detected in human ovarian carcinoma. The low expression of miR-199a has been previously detected in ovarian carcinoma and is significantly correlated with a poor prognosis (3). The purpose of the present study was to define the role of this miRNA during the development of cisplatin drug resistance in the human OV2008 and C13* ovarian cancer cell lines by analyzing the expression levels of miR-199a and mTOR, a possible target of miR-199a.

## Materials and methods

### Cell lines and culture

The cisplatin-resistant ovarian cancer cell line (C13*) and its sensitive variant (OV2008) were gifts from Dr Rakesh Goel at Ottawa Regional Cancer Center, Ottawa, Canada. These cell lines were maintained at 37ºC in RPMI-1640 complete medium supplemented with 2 mM L-glutamine and 10% fetal bovine serum in a humidified atmosphere of 5% CO_2_.

### Reagents and antibodies

Cisplatin and DMSO were purchased from Sigma Chemical Inc. (St. Louis, MO, USA). Fetal bovine serum, RPMI-1640, Lipofectamine 2000 reagent and TRIzol™ reagent were purchased from Life Technologies Inc. (Carlsbad, CA, USA). Cell counting kit-8 (CCK-8) was purchased from Dojindo Molecular Technologies, Inc. (Kumamoto, Japan). Rabbit anti-human mTOR polyclonal antibody was obtained from Cell Signaling Technology Inc. (Danvers, MA, USA) and β-actin antibody was obtained from Santa Cruz Biotechnology, Inc. (Santa Cruz, CA, USA). Luciferase reporter vectors were obtained from Promega Corporation (Madison, WI, USA) and PCR primers were obtained from Invitrogen Corporation (Carlsbad, CA, USA).

### miRNA transfection

The miR-199a mimics and inhibitors were purchased from Ambion (Life Technologies Inc.). OV2008 and C13* cells in the exponential phase of growth were plated in six-well plates at 3.5×10^5^ cells/well and cultured for 16 h. The cells were then transfected with the mimics or inhibitors of miR-199a or the negative control (NC) RNA, at a final concentration of 100 nM using Lipofectamine 2000 (Invitrogen) and OPTI-MEM reduced serum medium (Life Technologies Inc.), according to the manufacturer’s instructions. To determine the expression of mTOR, at 48 h post-transfection, the transfected cells were collected to measure the mRNA and protein levels.

### Quantitative (q)PCR for miR-199a and mTOR mRNA detection

Total RNA was extracted from cultured OV2008 and C13* cells according to the TRIzol-chloroform protocol and reverse transcribed into cDNA using M-MLV reverse transcriptase (Promega) and oligo(dT). The Bulge-Loop™ miRNA qPCR primer set for hsa-miR-199a (MQP-0101; RiboBio, Guangzhou, China) and U6 snRNA (MQP-0201; RiboBio) were used according to the manufacturer’s instructions. The cDNA was used for the amplification of mature miR-199a, mTOR, GAPDH and U6 snRNA through qPCR. The primer sequences of the mTOR and GAPDH were as follows: mTOR forward, 5′-AGGCCGCATTGTCTCTATCAA-3′ and reverse, 5′-GCAGTAAATGCAGGTAGTCATCCA-3′; and GAPDH forward, 5′-GTCAGTGGTGGACCTGACCT-3′ and reverse, 5′-AGGGGAGATTCAGTGTGGTG-3′. For the reverse transcription, 500 ng total RNA was transcribed into cDNA in a 20 μl reaction volume at 42ºC for 45 min with the GeneAmp Gold RNA PCR Reagent kit (Applied Biosystems, Foster City, CA, USA). qPCR was performed in a 20 μl reaction volume containing 10 μl SYBR Green PCR Master Mix (Applied Biosystems). The cycle conditions were 95ºC for 3 min, followed by 40 cycles of 95ºC for 20 sec, 60ºC for 30 sec and 70ºC for 30 sec. The relative miRNA levels of the samples from each cell line in each group were calculated using the 2^−ΔΔCt^ method.

### Western blot analysis

The cells were harvested and homogenized with lysis buffer at 48 h post-transfection. Proteins were resolved in an SDS/PAGE gel and transferred onto PVDF membranes, then subjected to immunoblot analysis using polyclonal antibodies against mTOR and β-actin. All antibodies were used at 1 μg/ml working concentration in PBS with 5% skimmed milk. The membrane was incubated with anti-mTOR and anti-β-actin antibodies separately overnight at 4ºC. Subsequent to washing the membrane with TBST, it was incubated with horseradish peroxidase (HRD)-conjugated rabbit secondary antibody. Specific proteins were visualized using enhanced chemiluminescence following the manufacturer’s instructions (Pierce Biotechnology, Inc., Rockford, IL, USA). Then, the blots were exposed to X-ray film to obtain optimal bands. The bands were quantified by using Image J software (National Institutes of Health, Bethesda, MD, USA).

### Construction of vector and luciferase reporter assay

The 3′-untranslated region (UTR) fragments of the mTOR gene were predicted to be complementary to the sequence of miR-199a according to an analysis of the miRNA target gene prediction database, TargetScan. The whole sequence of the mTOR 3′-UTR was amplified by PCR using human genomic DNA as a template. The primers for the 3′-UTR segment were 5′-CTGGAGGCCCAGATGTGCCCATCACG-3′ (sense) and 5′-ACATATGTTTAAAATTCTGATGTCAT-3′ (antisense). The PCR product was ligated into the PGM-T vector (Tiangen Biotech, Beijing, China). The mTOR 3′-UTR inserts were removed from the PGM-T plasmid and cloned downstream of the Renilla luciferase reporter gene (psiCHECK-2™; Promega). The accuracy of the inserted gene was confirmed by sequencing. At 24 h prior to transfection, the cells were plated at 5×10^5^ cells/well into 96-well plates. Luciferase 3′-UTR-reporter vectors (100 ng) and 100 nmol miR-199a mimics were co-transfected into C13* cells using Lipofectamine 2000 reagent according to the manufacturer’s instructions (Invitrogen). At 24 h post-transfection, the cells were harvested and lysed with passive lysis buffer (Promega). Luciferase activity assays were performed using the Dual Luciferase Reporter Assay System (Promega) following the manufacturer’s instructions. Three independent experiments were performed in triplicate.

### Cell viability measured by CCK-8 assay

The cytotoxic effects of cisplatin were determined with the CCK-8 assay. The cells were seeded in triplicate in 96-well plates the day prior to the experiment at a density of 5×10^5^ cells/well. Subsequent to 24 h, the OV2008 and C13* cells were transfected with the inhibitors or mimics of miR-199a for 24 h. Following 12 h of incubation, the cells were then treated with various concentrations of cisplatin for 48 h. The absorbance at 450 nm was measured using a multilabel plate reader (Perkin-Elmer, Waltham, MA, USA). The results are presented as the mean ± SD of three separate experiments, with six determinations per experiment.

### Apoptosis assay

At 24 h post-transfection, as described previously, the OV2008 and C13* cells were treated with cisplatin at a concentration of 40 μM for 48 h. The cells were washed twice with cold 10 mM PBS and resuspended in 1X binding buffer (BD Biosciences, San Jose, CA, USA) at a concentration of 1×10^6^ cells/ml. The cells were stained with 5 μl Annexin V and 10 μl propidium iodide (PI), using the Annexin V apoptosis detection kit (KeyGen Biotech, Nanjing, China) for 20 min at room temperature in the dark. The analysis of the apoptotic cells was performed with a FACScan (BD Biosciences) and the data were analyzed using CellQuest version 3.3 software (BD Biosciences). The experiment was repeated three times.

### Statistical analysis

All experiments were repeated at least three times. Numerical data are presented as the mean ± SD. P<0.05 was considered to indicate a statistically significant difference.

## Results

### Expression levels of miR-199a in OV2008 and C13* ovarian cancer cells

**Figure 1:**
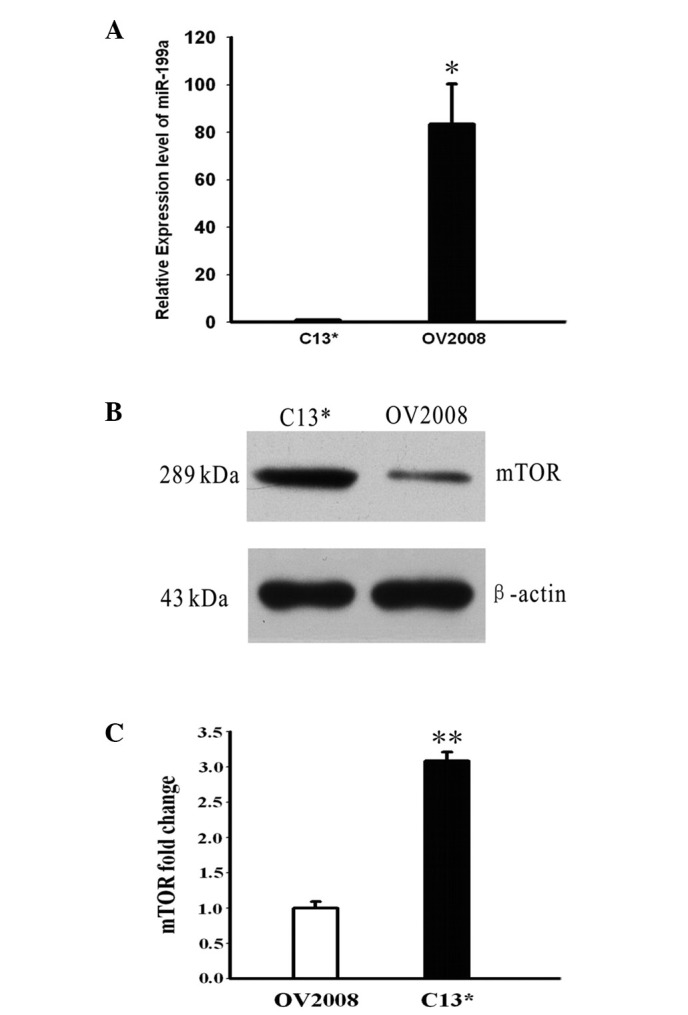
Expression levels of miR-199a and mTOR in OV2008 and C13* cells were detected by qPCR and western blotting. (A) Expression levels of miR-199a were, on average, 83.4-fold higher in the OV2008 cells compared with the C13* cells (P<0.05). (B) Noticeably more mTOR protein was expressed in the C13* cells compared with the OV2008 cells, as shown by western blotting. (C) The mTOR fold change in protein level is shown from three independent experiments. ^*^P<0.05 vs. C13*; ^**^P<0.05 vs. OV2008. mTOR, mammalian target of rapamycin.

The levels of miR-199a and mTOR were detected by qPCR and western blotting in the OV2008 and C13* cells. The expression levels of miR-199a were, on average, 83.4-fold higher in the OV2008 cells compared with the C13* cells (P<0.05, Fig. 1A). As shown in Fig. 1B, the expression of mTOR was noticeably higher in the C13* cells compared with OV2008 cells, as demonstrated by western blotting.

### Effect of miR-199a on sensitivity to cisplatin treatment in C13* and OV2008 cells

**Figure 2:**
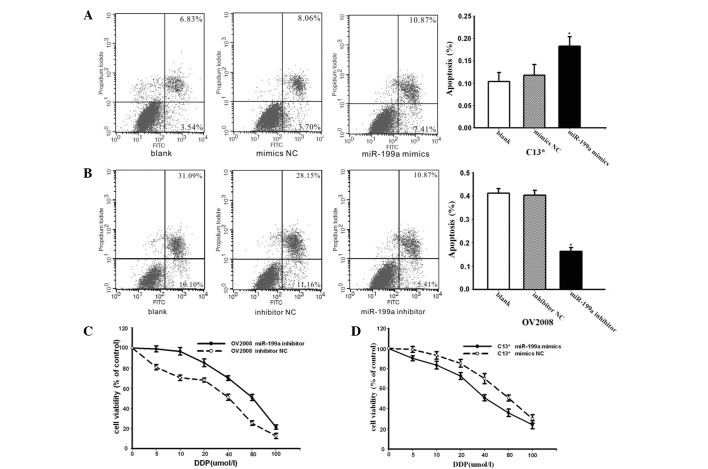
Effect of miR-199a on sensitivity to cisplatin treatment in C13* and OV2008 cells. (A) C13* cells were transfected with miR-199a mimics and miR-mimic negative control (NC), then treated with 40 μM cisplatin for 24 h. Cell apoptosis was measured by flow cytometry. (B) OV2008 cells were transfected with miR-199a inhibitor and inhibitor NC, then treated with 40 μM cisplatin for 24 h. Cell apoptosis was measured by flow cytometry. (C) Cisplatin sensitivity was decreased in miR-199a inhibitor-treated OV2008 cells compared with those treated with NC, and the viability of the cells was evaluated by the CCK-8 assay. (D) C13* cells transfected with mimics of miR-199a exhibited increased sensitivity to cisplatin treatment; the viability of the cells was evaluated by the CCK-8 assay. ^*^P<0.05 vs. NC. mTOR, mammalian target of rapamycin; CCK-8, cell counting kit-8.

The effect of miR-199a was further investigated in the cisplatin-resistant ovarian cancer cell line C13* through treatment with cisplatin. The C13* cells were transfected with miR-199a mimics and miR-mimic negative controls (NCs) and treated with of 40 μM cisplatin for 24 h. Apoptosis assays using annexin V staining indicated that the mimics of endogenous miR-199a enhanced cisplatin-induced apoptosis compared with the NC group (Fig. 2A). As shown in Fig. 2B, the OV2008 cells were transfected with miR-199a inhibitor or inhibitor NC, followed by treatment with 40 μM cisplatin for 24 h. Apoptosis assays using annexin-V staining showed that significantly lower apoptosis ratios were detected in the OV2008 cells transfected with miR-199a inhibitor compared with the NC group (Fig. 2B). These results indicated that miR-199a is able to reverse cisplatin-resistance in ovarian cancer cells by promoting cisplatin-induced apoptosis *in vitro*.

To further demonstrate whether miR-199a was able to regulate the sensitivity of OV2008 and C13* cells to cisplatin, the OV2008 and C13* cells were transfected with inhibitors of miR-199a or mimics of miR-199a, respectively. The cells were then incubated with cisplatin at various concentrations and the viability of cells was evaluated using the CCK-8 assay. As shown in Fig. 2C, transfection with inhibitors of miR-199a markedly decreased the sensitivity of the OV2008 cells to cisplatin compared with the cells treated with NC. The C13* cells transfected with mimics of miR-199a exhibited increased sensitivity to cisplatin compared with the cells treated with NC (Fig. 2D). These results clearly indicate that miR-199a is significant in the cisplatin resistance mechanism of ovarian cancer cells.

### Regulation of mTOR expression by miR-199a

**Figure 3:**
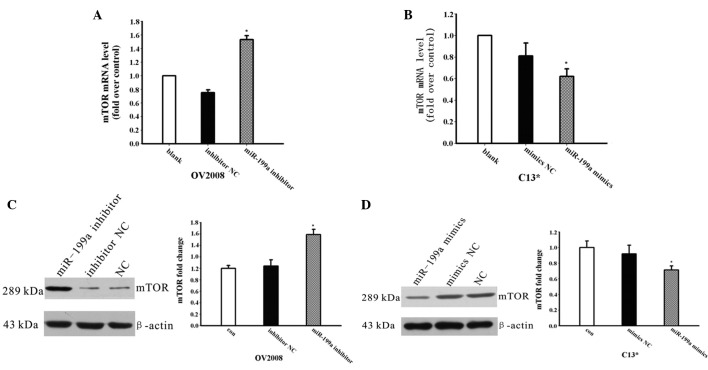
Regulation of mTOR expression by miR-199a. (A) Expression of mTOR mRNA in OV2008 cells transfected with inhibitor of miR-199a. (B) Expression of mTOR mRNA in C13* cells transfected with mimics of miR-199a. (C) Expression of mTOR protein in OV2008 cells transfected with inhibitor of miR-199a. (D) Expression of mTOR protein in C13* cells transfected with mimics of miR-199a. ^*^P<0.05 vs. NC. mTOR, mammalian target of rapamycin.

To investigate whether miR-199a is involved in the regulation of the expression of mTOR, the mimics or inhibitors of miR-199a were transfected into the C13* and OV2008 cells, respectively, and the mRNA and protein expression levels of mTOR were detected by qPCR and western blotting. As shown in Fig. 3A, the expression of mTOR mRNA was increased following miR-199a inhibitor transfection in the OV2008 cells. The mTOR protein expression level was also increased (Fig. 3C), while the mRNA and protein levels of mTOR were decreased in the C13* cells transfected with miR-199a mimics compared with the NC groups (Fig. 3D).

### mTOR may be a target gene of miR-199a

**Figure 4:**
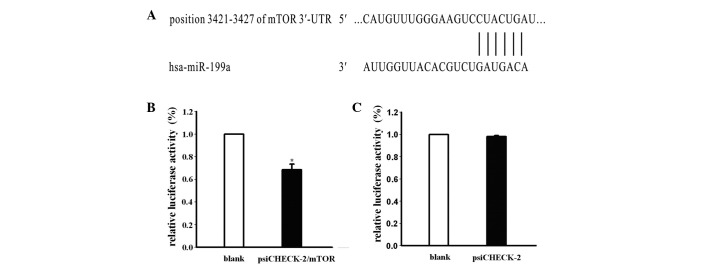
mTOR may be a target gene of miR-199a. (A) mTOR’s 3′-UTR mRNA includes a highly-conserved binding site for miR-199a. (B) C13* cells were co-transfected with psiCHECK-2™/mTOR 3′-UTR and miR-199a mimics or control oligonucleotides. At 24 h post-transfection, mTOR fluorescence intensity was detected. (C) Luciferase activity was not different between empty vector psiCHECK-2 and controls. ^*^P<0.05 vs. blank. mTOR, mammalian target of rapamycin; UTR, untranslated region.

Based on the present data, we attempted to identify whether mTOR is the target gene of miR-199a, which may explain miR-199a-related cisplatin resistance in ovarian cancer cells. Subsequent to analyzing miRNA target prediction public databases (TargetScan, Pictar), it was observed that the 3′-UTR mRNA of mTOR included a highly-conserved binding site for miR-199a (Fig. 4A). To investigate the association between mTOR and miRNA, the C13* cells were co-transfected with mimics of miR-199a and vector containing a psiCHECK-2 Renilla luciferase reporter gene and the 3′-UTR mRNA of mTOR or empty vector psiCHECK-2. mTOR fluorescence intensity was detected at 24 h post-transfection. The results showed that the luciferase activity of the psiCHECK-2 Renilla luciferase reporter gene with the 3′-UTR mRNA of mTOR was significantly decreased by 68.4%, but that there was no difference in luciferase activity between the empty vector psiCHECK-2 and controls (Fig. 4B and C). These results indicated that mTOR expression was significantly blocked by miR-199a. Consequently, mTOR may be the target gene of miR-199a.

## Discussion

Aberrant miRNAs are capable of affecting the expression of target proteins, which may affect cell death signaling pathways, drug targets or cell cycle-related proteins. This may lead to altered resistance to cytotoxic therapy (4). Academic conferences have paid close attention to miRNAs (5). Currently, the main obstacle to successful chemotherapy is the development of drug resistance to chemotherapeutics. The defective apoptosis pathway is a major mechanism of drug resistance in ovarian cancer cells. Increasing evidence indicates that miRNAs are involved, at least partially, in the drug resistance of ovarian cancer cells, through this mechanism (6).

Restoring attenuated levels of miR-199a in human hepatocarcinoma cells results in G_1_-phase cell cycle arrest, leading to reduced invasive ability, increased susceptibility to hypoxia and enhanced sensitivity to doxorubicin-induced apoptosis (7). These characteristics make miR-199a a biomarker of hepatocarcinoma (8). According to Sorrentino *et al*, miRNAs may play a significant role in drug resistance by targeting different genes in different cancer cell lines (6). Based on previous research, miR-199a may be a potential cancer suppresser and could act as a new therapeutic target for ovarian cancer patients with a risk for cisplatin resistance.

In the present study, the expression levels of miR-199a were analyzed in OV2008 and C13* cells and attempts were made to identify the molecular mechanism between cisplatin resistance and miR-199a. First, it was demonstrated that the miR-199a level was lower in the C13* cells compared with the parental OV2008 cells, while the expression of mTOR was noticeably higher. Subsequently, inhibitors of miR-199a were transfected into the OV2008 cells and it was observed that the miR-199a reduction increased the mTOR expression level, decreasing the sensitivity of the OV2008 cells to cisplatin. Transfecting mimics of miR-199a into the C13* cells caused the expression level of mTOR to be reduced, while the sensitivity to cisplatin was increased. These results show that miR-199a is able to reverse cisplatin chemoresistance by the negative regulation of mTOR expression. It is known that the effect of miRNAs depends on the process of post-transcriptional gene silencing. Consequently, miRNAs may inhibit certain transcription factors that are associated with the translation of mTOR during this process.

To identify specific mRNA functional fragments in miRNA, various algorithms for the target prediction were tested, including those used in the TargetScan, PicTar and miRanda web sites. The intersection of algorithms indicated that mTOR was a potential target gene of the mature miR-199a. mTOR has been demonstrated to be a crucial kinase acting downstream of the activation of the PI3K signaling pathway. Evidence indicates that mTOR functions as a master switch of cellular catabolism and anabolism, thus determining whether cancer cells grow and proliferate (7). Moreover, mTOR has been demonstrated to have marked effects on the modulation of apoptotic cell death, which primarily depends on the cellular context and downstream signaling proteins, including p53, B-cell lymphoma (BCL2), p21, p27 and c-MYC (9). mTOR inhibition restores sensitivity to certain existing chemotherapeutic agents such as cisplatin, trastuzumab and gefitinib. The molecular mechanisms leading to apoptosis in tumor cells have not been fully understood. One possible association between mTOR inhibition and apoptosis induction may be provided by the downstream target S6K1, which phosphorylates the pro-apoptotic molecule BCL2-antagonist of cell death (BAD) on Ser136, a reaction that disturbs the binding of BAD to the mitochondrial death inhibitors BCL-XL and BCL2, thus inactivating BAD (10). In this case, rapamycin-mediated S6K1 inactivation would indirectly cause BAD activation. Moreover, several growth factors that activate the PI3K and S6K1 signaling pathway were recently shown to increase the expression of BCL2, thus promoting cell survival in myeloid progenitor cells (11). Studies in cancer cells have indicated that BCL2 contributes to chemotherapy resistance, and the aberrant expression of BCL2 has been associated with drug resistance to commonly used anticancer agents (12).

Tsurutani *et al*(13) demonstrated that LY294002 and imatinib caused greater than additive increases in apoptosis compared with apoptosis caused by the inhibitor or imatinib alone. This result indicated that laminin-mediated activation of the PI3K/AKT/mTOR signaling pathway was a mechanism of cellular survival and therapeutic resistance in small cell lung cancer cells. CCI-779, a known mTOR inhibitor, is able to reverse cisplatin resistance (2) and mTOR may be involved through the following possible mechanism: Active AKT results in the activation of multiple downstream effectors that combine with mTOR to increase the translation of proteins essential for survival. The inhibition of mTOR enhances the sensitivity of a broad range of chemocytotoxic agents, including cisplatin, carboplatin, doxorubicin, mitoxantrone and doxcetaxel in numerous types of human cancers. RAD001 is an mTOR inhibitor. RAD001 in combination with cisplatin was shown to induce a distinct increase in the number of apoptotic cells by downregulating the pro-survival molecules, BCL2, survivin and cyclinD1, compared with RAD001 or cisplatin alone. RAD001 enhanced the sensitivity of hepatocellular carcinoma cells to cisplatin in the p53-dependent and -independent pathways (14). Certain inhibitors of PI3K/mTOR have been involved in clinical trials (15).

The AKT/mTOR signaling pathway has a major role in cisplatin resistance in ovarian cancer cells; LY294002, the inhibitor of PI3K/mTOR, has been shown to sensitize ovarian cancer cells to cisplatin (16). Another study indicated that the expression of the PI3K-p85 subunit was higher in epithelial ovarian cancer specimens at the protein level, but that it was not detected in the normal ovarian epithelium (17). In the chemoresistant ovarian cancer cells, SKOV3/DDP and SKOV3/MCA, elevated activation of the AKT/mTOR/survivin signaling was observed. Downregulation of the mTOR/survivin signaling pathway attenuated cisplatin resistance (17). The mTOR signaling pathway may be involved in regulating the phosphorylation status of p70S6K at Ser371 in the mediation of chemoresistance in ovarian cancer (18).

In summary, the present study indicates that miR-199a contributes to the reversal of cisplatin resistance by blocking the expression of mTOR in cisplatin-resistant ovarian cancer cells. During this process, mTOR is at least an indirect target gene of miR-199a. Future studies are required to further demonstrate whether mTOR is a direct target of miR-199a and whether additional molecular mechanisms exist (19). According to the present study results, we predict that miR-199a may be a potential therapeutic target for cisplatin-resistant ovarian cancer.
